# Bayesian Geostatistical Analysis and Ecoclimatic Determinants of *Corynebacterium pseudotuberculosis* Infection among Horses

**DOI:** 10.1371/journal.pone.0140666

**Published:** 2015-10-16

**Authors:** Courtney Boysen, Elizabeth G. Davis, Laurie A. Beard, Brian V. Lubbers, Ram K. Raghavan

**Affiliations:** 1 Department of Clinical Sciences, College of Veterinary Medicine, Kansas State University, Manhattan, Kansas, United States of America; 2 Kansas State Veterinary Diagnostic Laboratory, Department of Diagnostic Medicine and Pathobiology, Kansas State University, Manhattan, Kansas, United States of America; The University of Melbourne, AUSTRALIA

## Abstract

Kansas witnessed an unprecedented outbreak in *Corynebacterium pseudotuberculosis* infection among horses, a disease commonly referred to as pigeon fever during fall 2012. Bayesian geostatistical models were developed to identify key environmental and climatic risk factors associated with *C*. *pseudotuberculosis* infection in horses. Positive infection status among horses (cases) was determined by positive test results for characteristic abscess formation, positive bacterial culture on purulent material obtained from a lanced abscess (*n* = 82), or positive serologic evidence of exposure to organism (≥1:512)(*n* = 11). Horses negative for these tests (*n* = 172)(controls) were considered free of infection. Information pertaining to horse demographics and stabled location were obtained through review of medical records and/or contact with horse owners via telephone. Covariate information for environmental and climatic determinants were obtained from USDA (soil attributes), USGS (land use/land cover), and NASA MODIS and NASA Prediction of Worldwide Renewable Resources (climate). Candidate covariates were screened using univariate regression models followed by Bayesian geostatistical models with and without covariates. The best performing model indicated a protective effect for higher soil moisture content (*OR* = 0.53, 95% *CrI* = 0.25, 0.71), and detrimental effects for higher land surface temperature (≥35°*C*) (*OR* = 2.81, 95% *CrI* = 2.21, 3.85) and habitat fragmentation (*OR* = 1.31, 95% *CrI* = 1.27, 2.22) for *C*. *pseudotuberculosis* infection status in horses, while age, gender and breed had no effect. Preventative and ecoclimatic significance of these findings are discussed.

## Introduction

*Corynebacterium pseudotuberculosis* is a pleomorphic, gram-positive, facultative intracellular bacterium with a worldwide distribution that causes disease in cattle, sheep, goats and horses [1] [2]. The bacterium is soil-borne, and horses are believed to acquire it from inhaling infected soil or through contamination of abraded skin, and also through biting flies [1]. The bacterium causes three clinical syndromes in horses, the most prevalent of which is the formation of external abscesses that contain abundant purulent material and are characteristically located in the pectoral region, ventral abdomen, sheath, or mammary gland, which is commonly referred to as pigeon fever, pigeon breast or dryland distemper. The second clinical syndrome occurs less frequently (<10% of cases), as an internal infection with development of either single or multiple abscesses in the abdominal or thoracic cavity. Although internal infections are rare, a reported mortality risk of 40% is seen even with appropriate and aggressive antimicrobial treatment, and in the absence of treatment mortality rate increases to 100% [2]. The third and least common manifestation of disease associated with *C*. *pseudotuberculosis* is ulcerative lymphangitis.

Infection with *C*. *pseudotuberculosis* is traditionally considered to be an arid region disease perhaps due to its first identification among horses in drier areas of California, where the disease is now enzootic. However, outbreaks of this disease outside this region have been recorded periodically [2], [3]. Potential environmental or latent climatological influences associated with these outbreaks are seldom reported. Large outbreaks in the Midwestern US such as the one Kansas witnessed in 2012 are so far rare. However, the number of cases seen within this region, particularly in Texas has been increasing over the years, indicating a plausible eastward expansion of this disease’s enzootic range. Between the years 2005–2011, the number of confirmed cases tested at Texas Veterinary Medical Diagnostic Laboratory (TVMDL) increased an annual average of 177% [4], [5]. Most of these cases clustered around the panhandle and Central Texas regions, which experience semi-arid to subtropical climate, unlike the epizootic arid west. The 2012 outbreak in Kansas also occurred in areas that received relatively higher amounts of precipitation but followed an exceptionally warm winter and hot and dry summer seasons that year [6], [7].

While there is a chance for new infections to occur outside the epizootic areas through contact with horses that were infected elsewhere, it is more or at least equally likely for the infections to originate locally from *C*. *pseudotuberculosis* that may be present in soils and/or other hosts. This is often suspected to be the source of infection for horses that have had no apparent contact with travelling or infected horses. Information on the ecology and spatial distribution of the pathogen is however lacking. Environmental and climatological influences such as a prolonged drought could trigger infections in new areas [5], [8]. Understanding such influences may have important preventative and management implications, and as well as help recognize the eco-climatological influences in the ecology and evolution of this disease. Doherr et al. [9] reported that young adult horses and those that had contact with other horses on summer pastures were at significant risk for pigeon fever in California. Besides this study, evaluations of environmental or climatological risk factors for *C*. *pseudotuberculosis* infection, which could vary over geographic regions cannot be found. Soil types and conditions such as moisture levels, and *p* ^*H*^ could affect the survival and *C*. *pseudotuberculosis* infectivity, while a horse’s exposure to extreme climatic events and different ground cover or landscape patterns that support reservoir hosts could increase risk of infection.

In this study, we evaluated associations of such potential risk factors for *C*. *pseudotuberculosis* infection among horses that were seen between 2005–2013 period in Kansas and neighboring states. For this, we used medical records of horses that had a positive or negative diagnosis for *C*. *pseudotuberculosis* infection that were received at the Kansas State University’s Veterinary Health Center (VHC) and Kansas State Veterinary Diagnostic Laboratory (KSVDL), and publicly available environmental and climatological covariate data. Following univariate screening of candidate variables, models were constructed in a Bayesian hierarchical framework, which has advantages over traditional methods when there is a potential for spatial influence on disease data [10], [11].

## Materials and Methods

### 2.1 Study region

**Fig 1:**
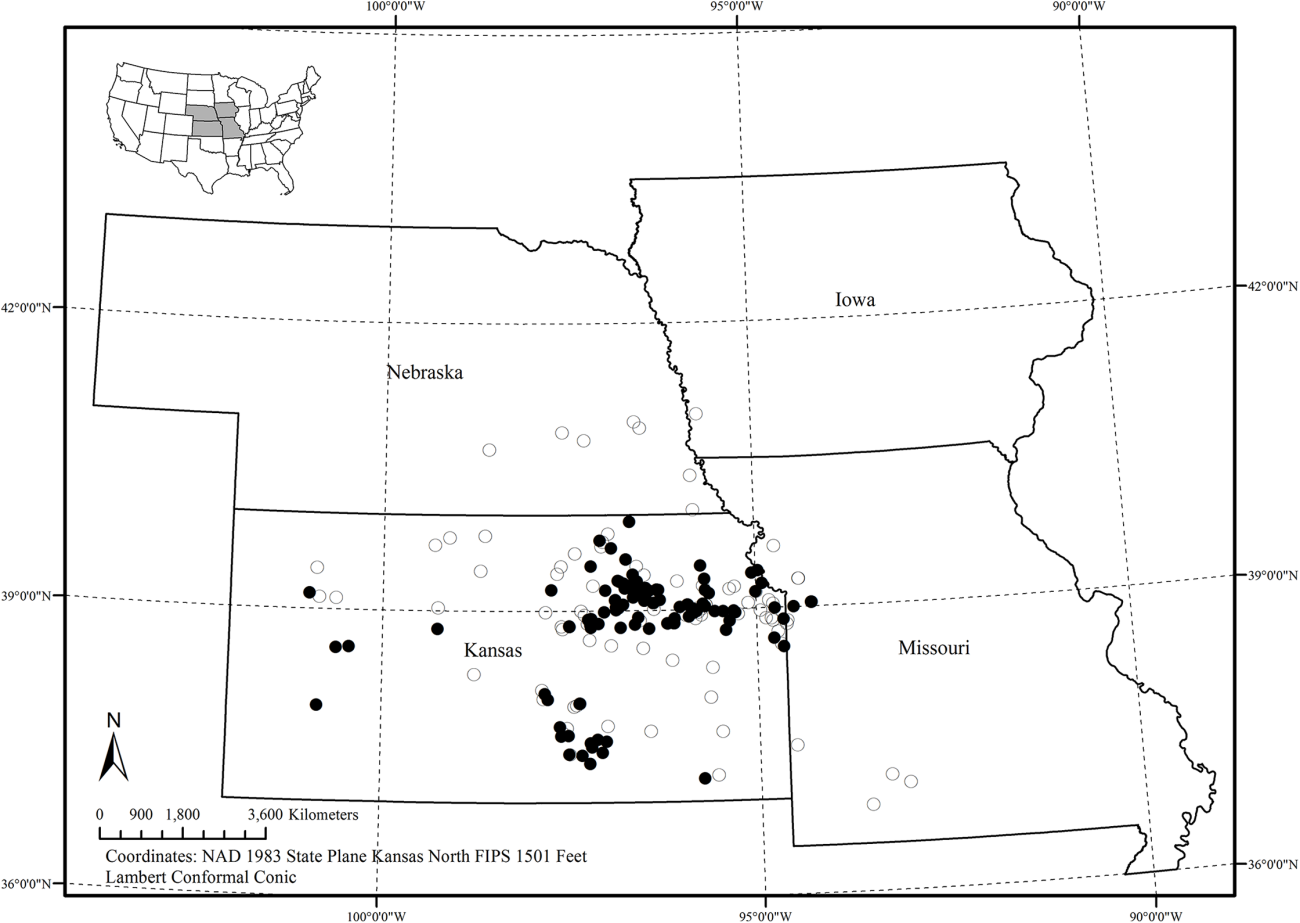
Case control distribution in the study region. Dark circles indicate case locations and open circles are control locations.

Most cases reported to VHC and KSVDL during the 2012 outbreak originated from Kansas, and few were from neighboring Nebraska and Missouri (Fig 1). With a mid-latitude and continental location, temperature extremes in Kansas occur in July (26°*C*) and January (−1.6°*C*) (30 year averages). Over 70% of the precipitation occurs during the warm season, with a peak in June (105 mm) and the minimum in January (18 mm) (30 year averages) [12]. There are large variations in the yearly precipitation received across the state of Kansas, with eastern Kansas receiving up to three times more rainfall than west [13]. As a result, climate and vegetation are transitional between the humid east and semi-arid western portions of Kansas.

### 2.2 Data

#### 2.2.1 Ethics statement

This study did not involve human subjects. No patient owner names were recorded or used during the completion of this study to maintain patient owner confidentiality and to adhere to the International Ethical Guidelines for Biomedical Research Involving Human Subjects. All patient owner records/information was anonymized and de-identified prior to analysis.

#### 2.2.2 Epidemiological data

All medical records entered between January 2005 and April 2013 were searched from the VHC and KSVDL databases that had keywords typically associated with pigeon fever infection in horses (Equine; *Corynebacterium*; *pseudotuberculosis*; pigeon fever; pectoral; abscess). A total of 317 unique records were retrieved and the owner’s address at the time infection was diagnosed were digitized, out of which 32 (10.0%) had business addresses provided and were therefore removed. On further spatial evaluation of the remaining records using land cover/land use data [14] 31 (8.6%) records revealed that the land within 2.5 km area surrounding their addresses contained built environment, suggesting that the horses had lived elsewhere and were therefore removed. From the remaining 254 records all information pertaining to the diagnosis (presence or absence of *C*. *pseudotuberculosis* infection in horses), horse demographics (age, sex, and breed), and season of case presentation (spring = March–May, summer = June–August, Fall = September–November, Winter = December–February) was digitized. Horses were grouped into eight categories by 5-year ages groups (<1, 1 to 6, 6 to 11, 12 to 17, 18 to 23, 24 to 29, >29, and Unknown) and by sex into four groups (Male, Female, Male neutered, and Unknown) (Table 1).

**Table 1:** Case-control characteristics enrolled in the study.

	Case n (%)	Control n (%)
***Age (years)*:**		
<1	2 (2.44)	17 (9.88)
1 to 6	14 (17.07)	34 (19.77)
7 to 11	22 (26.83)	44 (25.58)
12 to 17	24 (29.27)	28 (16.28)
18 to 23	11 (13.41)	18 (10.47)
24 to 29	5 (6.10)	4 (2.33)
29 and more	1 (1.22)	5 (2.91)
Unknown	3 (3.66)	22 (12.79)
***Sex*:**		
Male	9 (10.97)	26 (15.11)
Female	39 (47.56)	65 (37.79)
Male neutered	28 (34.14)	64 (37.20)
Unknown	6 (7.31)	17 (9.88)

The inclusion criteria employed for selecting cases and controls that were enrolled in this study is as following. Medical records that indicated positive bacterial culture for *C*. *pseudotuberculosis* on purulent material obtained from abscess and/or a positive titer (≥ 512) on synergistic hemolysis-inhibition titer (SHI) test [15] were considered to confirm positive infection status in horses. Among horses that were presented at VHC, those that had initially displayed characteristic abscess formation (n = 25) were tentatively determined to be positive for *C*. *pseudotuberculosis* infection. Positive bacterial culture on purulent material obtained from abscess further confirmed infection status among these horses. Horses received at VHC that show symptoms of respiratory disease or systemic inflammatory disease that are consistent with pigeon fever but without visible external abscess are routinely referred for screening using SHI test. During the study period, horses that met these criteria (n = 21) were referred for SHI testing, and 14 were later confirmed positive based on an SHI titer ≥ 1:512. In two of these cases horses had both a positive culture and an SHI titer ≥ 1:512. Among horses that were referred for diagnostic testing at KSVDL, case records that indicated positive bacterial culture for *C*. *pseudotuberculosis* from purulent material obtained from abscess (n = 26) and SHI titer ≥ 1:512 (n = 17) confirmed pigeon fever status in horses. The above inclusion criteria resulted in a total of 82 cases.

The inclusion criteria for controls used medical records, queried using the same keywords as above that indicated negative bacterial culture for *C*. *pseduotuberculosis* from purulent material obtained from abscess (n = 45), SHI titers ≤ 512 (n = 7, VHC; n = 29, KSVDL), and determination of other etiological causes during necropsy (n = 45) and cytologic examinations (n = 9), routine biopsy (n = 16) and clinical evaluations (n = 19, VHC). This resulted in the inclusion of 172 controls for this study.

#### 2.2.3 Covariate data

Environmental covariates from three broad thematic groups were obtained from publicly available sources using Geographic Information Systems (GIS) and remote-sensing methods; soil attributes from USDA State Soil Geographic Survey (SSURGO) [16], land use/land cover attributes from the USGS National Land Cover Dataset (NLCD) [14], and climate data from NASA Moderate Resolution Imaging Spectroradiometer (MODIS) [17] and Prediction of Worldwide Renewable Resources (POWER) [18]. Covariate values from these geographic datasets were obtained by constructing 2.5 km buffer areas surrounding locations where horses had resided prior to their diagnosis. A list of all variables evaluated in this study is present in Table 2. Two landscape metrics were derived from the NLCD. The total edge contrast index (*TECI*) were estimated using Fragstats 4.0 [19] by,

$$TECI=\left[\frac{\overset{m}{\underset{i=1}{\Sigma }}\overset{m}{\underset{k=i+1}{\Sigma }}e_{ik}d_{ik}}{E^{*}}\right](100).$$

where *e*_*ik*_ is the total length of edge between patch types *i* and *k*, *E* ^*^ is the total length of edge in landscape, *d*_*ik*_ is the dissimilarity (edge contrast weight) between patches *i* and *k*; and, largest patch index (*LPI*) was calculated by,

$$LPI=\left[\frac{\overset{n}{\underset{j=1}{\Sigma }}a_{ij}}{A}\right]$$

Where *a*_*ij*_ is the area of patch *i*, *j* and *A* being total landscape area.

**Table 2:** Potential explanatory variables considered in the study.

Source/Variable	Control (Mean ± S.D)	Case (Mean ± S.D)	*p* ≤ 0.2
***USDA State Soil Geographic Survey (SSURGO)***			
Soil moisture	41.2 ± 10.5	20.8 ± 5.0	0.001
*p* ^*H*^	7.4 ± 0.5	7.3 ± 0.4	0.354
Organic matter content	7.3 ± 1.4	14.1 ± 5.2	0.258
Excessively drained	14 ± 3.2	13.1 ± 6.7	0.745
Somewhat excessively drained	8.1 ± 3.4	14.1 ± 7.2	0.621
Moderately well drained	17.0 ± 6.2	15.0 ± 8.1	0.662
Well drained	18.2 ± 4.6	17.0 ± 6.2	0.225
Poorly drained	5.6 ± 2.1	4.9 ± 2.1	0.548
Somewhat poorly drained	4.2 ± 1.2	3.8 ± 1.5	0.741
Frequent flooding	1.9 ± 0.9	2.3 ± 1.2	0.811
Rare flooding	1.8 ± 0.4	2.4 ± 1.0	0.514
Very rare flooding	4.2 ± 1.4	3.5 ± 1.5	0.332
No flooding	1.5 ± 0.7	2.3 ± 1.1	0.211
***USGS National Land Cover Dataset***			
*a*. *Percentage ground cover*			
Barren land	11.9 ± 8.3	8.2 ± 3.9	0.514
Deciduous forest	14.6 ± 5.6	17.2 ± 6.8	0.841
Mixed forest	2.6 ± 1.0	1.6 ± 0.9	0.153
Evergreen forest	11.2 ± 6.3	17.3 ± 8.7	0.354
Scrub/shrub	2.3 ± 0.8	4.2 ± 1.8	0.458
Grassland/herbaceous	28.4 ± 3.1	25.36 ± 2.8	0.122
Pasture/hay	14.6 ± 5.3	7.2 ± 3.4	0.224
Woody wetlands	8.6 ± 3.8	2.5 ± 0.9	0.541
Emergent herbaceous wetlands	4.6 ± 2.1	6.3 ± 3.1	0.365
*b*. *Landscape metrics*			
Total Edge Contrast (TECI) (habitat fragmentation)	37.5 ± 12.6	49.6 ± 8.0	0.008
Largest Patch Index (LPI)	12.8 ± 5.3	13.5 ± 4.9	0.367
***NASA Moderate Resolution Imaging Spectroradiometer (MODIS)***			
*a*. *Daytime land surface temperature*			
≥35°*C*	37.2 ± 1.7	39.4 ± 0.8	0.011
28−34.9°*C*	29.3 ± 0.8	29.1 ± 1.2	0.211
24.9−27.9°*C*	26.1 ± 1.1	25.9 ± 0.4	0.284
≤25°*C*	Reference category		
*b*. *Night-time land surface temperature*			
≤16°*C*	Reference category		
15.9−19.9°*C*	16.5 ± 1.1	16.7 ± 1.3	0.618
≥20°*C*	21.8 ± 0.9	22.1 ± 0.8	0.514
*c*. *Diurnal temperature range*			
	14.3 ± 0.3	14.1 ± 0.2	0.287
***NASA Prediction of Worldwide Renewable Resources (POWER)***			
Normalized Difference Vegetation Index (NDVI)	0.28 ± 0.1	0.31 ± 0.04	0.451
Daily maximum temperature	30.3 ± 1.4	31.4 ± 0.8	0.365
Daily minimum temperature	16.8 ± 1.4	17.8 ± 0.5	0.224
Daily average temperature	28.1 ± 2.1	29.0 ± 0.9	0.578
Dew point	61 ± 6.2	54 ± 8.1	0.741
Relative humidity	76 ± 10.2	80 ± 11.1	0.248
Diurnal temperature range	13.5 ± 0.4	13.7 ± 0.2	0.621

A total of 35 variables were considered for univariate evaluations. There were 14 variables derived from the USDA SSURGO database, 11 from NLCD, and 10 variables from NASA’s MODIS and POWER sources. All variables except daytime land surface temperature and night-time land surface temperature were in continuous form. ≤ 25°*C* and ≤ 16°*C* were used as reference categories in the models for daytime land surface temperature and night-time land surface temperature, respectively. A total of 5 variables retained significance in the univariate screening, with a liberal *p*−*value* ≤ 0.2. They were, soil moisture (*p* = 0.001), mixed forests (*p* = 0.153), grassland/herbaceous cover(*p* = 0.122), total edge contrast index (*p* = 0.008), and day time land surface temperature (≥ 35°*C*) (*p* = 0.011).

### 2.3 Statistical analysis

#### 2.3.1 Univariate covariate selection

Candidate covariates were screened in order to avoid convergence and mixing issues that result when numerous covariates are included in a model. Separate univariate logistic regression models evaluated each covariate independently and those that were significant at *p* ≤ 0.2 were kept. A logistic regression takes the form,

$$\text{log}\;\left[\frac{p_{i}}{1-p_{i}}\right]=\beta_{0}+\beta_{k}vk_{i}$$

Where *p*_*i*_ is presence of *C*. *pseudotuberculosis* infection, *β*_0_ the intercept coefficient, and *β*_*k*_ the coefficient for the explanatory variable *vk*_*i*_(*k* = 1,..,*n*). Non-linearity in covariate association with infection status in horses was checked and covariates with non-linear relationship were categorized based on diagnostic scatter plots. The variables, daytime land surface temperature and night time land surface temperature were categorized in to four and three categories, respectively for ease of interpretation despite their relationship with case status being linear. All other variables were kept in their original, continuous format. Multicollinearity among screened variables was tested by estimating the variance inflation factor (*VIF*) and all variables with a *VIF* ≥ 10 were considered to indicate multicollinearity [20], in which case, the least biologically relevant variable was dropped at a time until multicollinearity was absent. Two-way interaction terms were evaluated, and care was taken not to remove candidate variables that were deemed clinically relevant [20].

#### 2.3.2 Bayesian model specification

We assumed that infection status *Y*_*ij*_ of a horse *i* at location *S*_*j*_, which takes the value of 1 if the horse has positive diagnosis and 0 otherwise, followed a Bernoulli distribution, *Y*_*ij*_ ∼ *Ber*(*p*_*ij*_). The method is an extension of a GLM using the logit link function as follows:

$$\text{log}\;\left[\frac{p_{i}}{1-p_{i}}\right]=\beta_{0}+\beta_{1}v1_{i}+,\ldots ,+\beta_{k}vk_{i}+S_{i}$$

The stochastic spatial component *S*_*i*_ was modeled as a zero mean Gaussian process with variance *σ* ^2^ and autocorrelation function, *ρ*(*d*_*ij*_, *θ*), where *d*_*ij*_ = *x*_*i*_ − *x*_*j*_ measures the Euclidean distance between *x*_*i*_ and *x*_*j*_, and *θ* = [*ϕ*,*σ* ^2^,*τ* ^2^], where *ϕ* and *τ* are the decay and nugget parameters in a resulting semivariogram. The spatial component helps to account for the spatial variation in the residuals after model fit using covariates alone. The model parameters were estimated using a Bayesian framework with a Markov chain Monte Carlo (MCMC) algorithm in OpenBugs program [21] in a Beocat cluster computing environment [22]. Distributions of covariate effects on *C*. *pseudotuberculosis* prevalence are seldom available for the region; therefore non-informative, uniform priors were selected for the regression parameters, *β*_*k*_ and their variance component, $\sigma_{k}^{2}$. This allows the observed data to have the greatest influence on posterior distributions without being constrained by the choice of prior and can also improve MCMC convergence [23].

#### 2.3.3 Bayesian geostatistical modeling

Two Bayesian geostatistical models were fit to describe *C*. *pseudotuberculosis* infection status among horses. The first model included only the intercept and stochastic spatial component, *S*_*i*_, and a second model included the covariates screened in the univariate procedure as fixed effects along with *S*_*i*_. The reason for excluding/including the covariates was to determine if this had an effect on the model fit and to determine whether the covariates accounted for part or all of the spatial autocorrelation in pigeon fever data. For both models an initial burn-in of 10,000 iterations was allowed (later discarded), followed by 100,000 simulations after which the intercept (*α*), smoothing parameter (*κ*), decay parameter (*ϕ*) and covariate parameter estimate values were recorded. In order to determine whether sufficient iterations had been conducted to fully describe the posterior distributions, Monte Carlo error $\left(\frac{MCE}{SD}\right)$ was calculated for each variable and if $\left(\frac{MCE}{SD}\right)$ was ≤ 0.05, it was decided that sufficient iterations had been conducted [24]. For the second model, a full model with all covariates was fitted followed by removal of covariates one at a time. If the removal of a covariate resulted in higher DIC value (≥5) units we considered that to indicate poor performance and that variable was retained in the model. Previously removed covariates did not re-enter the final model. The median covariate estimates from the posterior distribution were calculated and exponentiated to provide odds ratios (ORs) along with their corresponding uncertainty measures (95% Bayes credible intervals). Potential confounders (age, sex, and breed) were inserted in the final Bayesian model one at a time and changes to DIC values and parameter estimates were recorded. If these additions resulted in ≥10% change to the posterior median estimates then those variables were retained in the model and adjusted ORs recorded.

## Results

**Fig 2:**
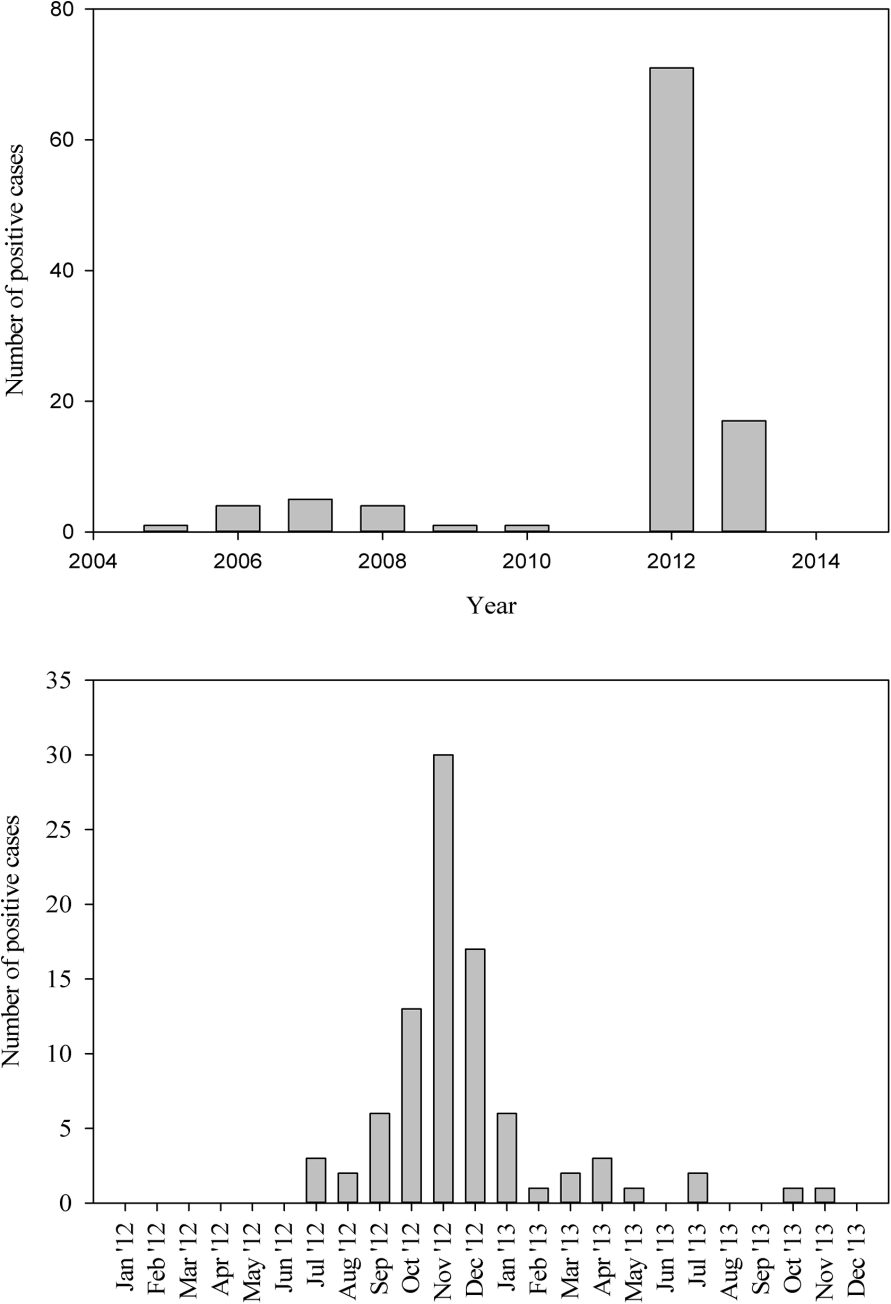
Number of positive pigeon fever cases diagnosed between 2005–2013 (a), and during 2012/13 (b) at the Veterinary Health Center and Kansas State Veterinary Diagnostic Laboratory, Kansas State University.

**Fig 3:**
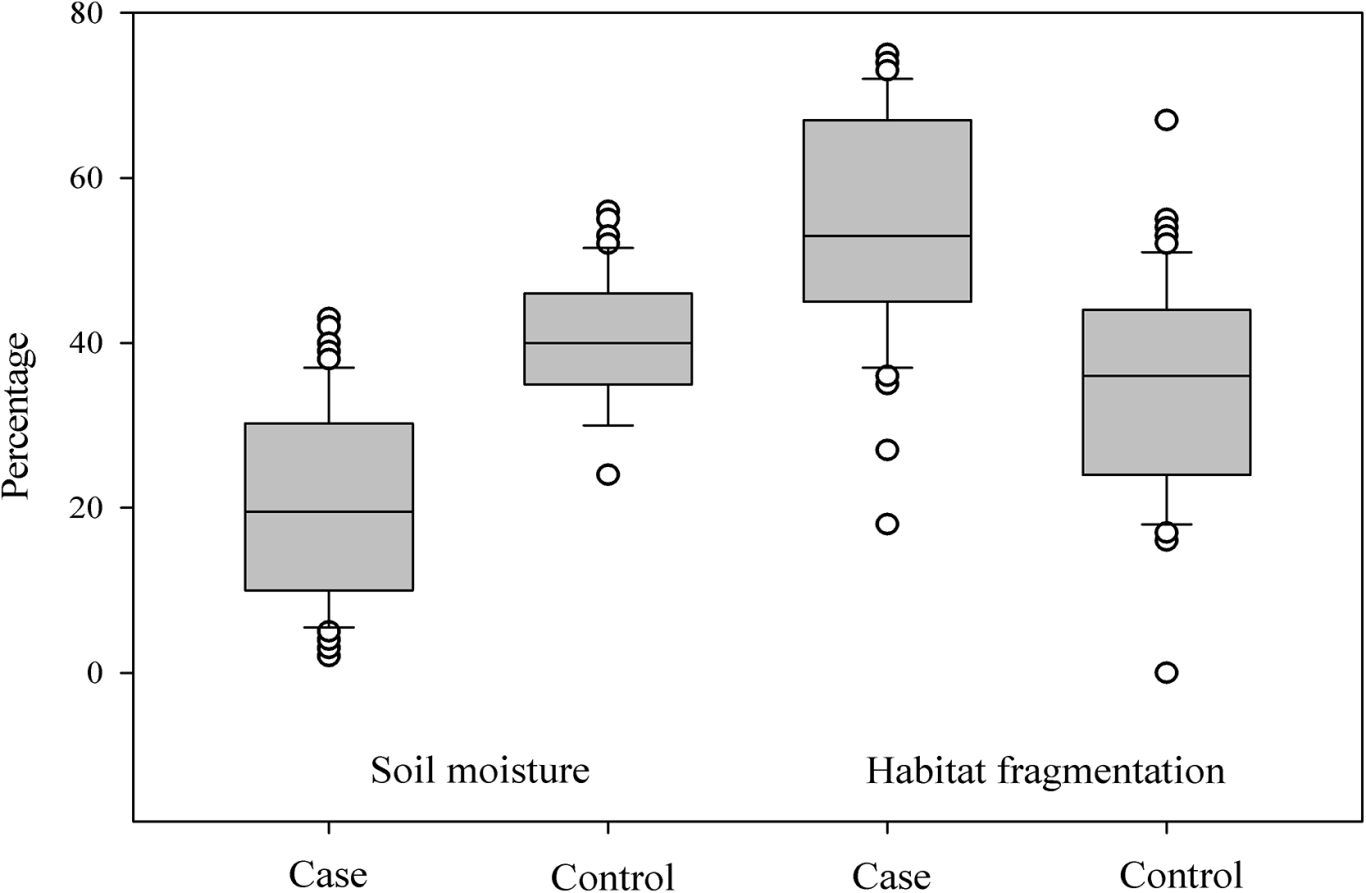
Distribution of percentage soil moisture and habitat fragmentation, surrounding case control locations in the study region. Soil moisture readings pertain to the soil moisture profile for a referenced map unit in a 2.5 km buffer area surrounding case control locations, recorded every month and averaged annually and describe the representative soil moisture situation for the map unit throughout the year [16]. Total edge contrast index, a measure of landscape fragmentation was measured by estimating the sum of lengths of each edge segment in the landscape multiplied by the corresponding contrast weight, divided by the total length of edge in the landscape and multiplied by 100 for percentages, for a 2.5 km buffer area surrounding case control locations [38].

**Fig 4:**
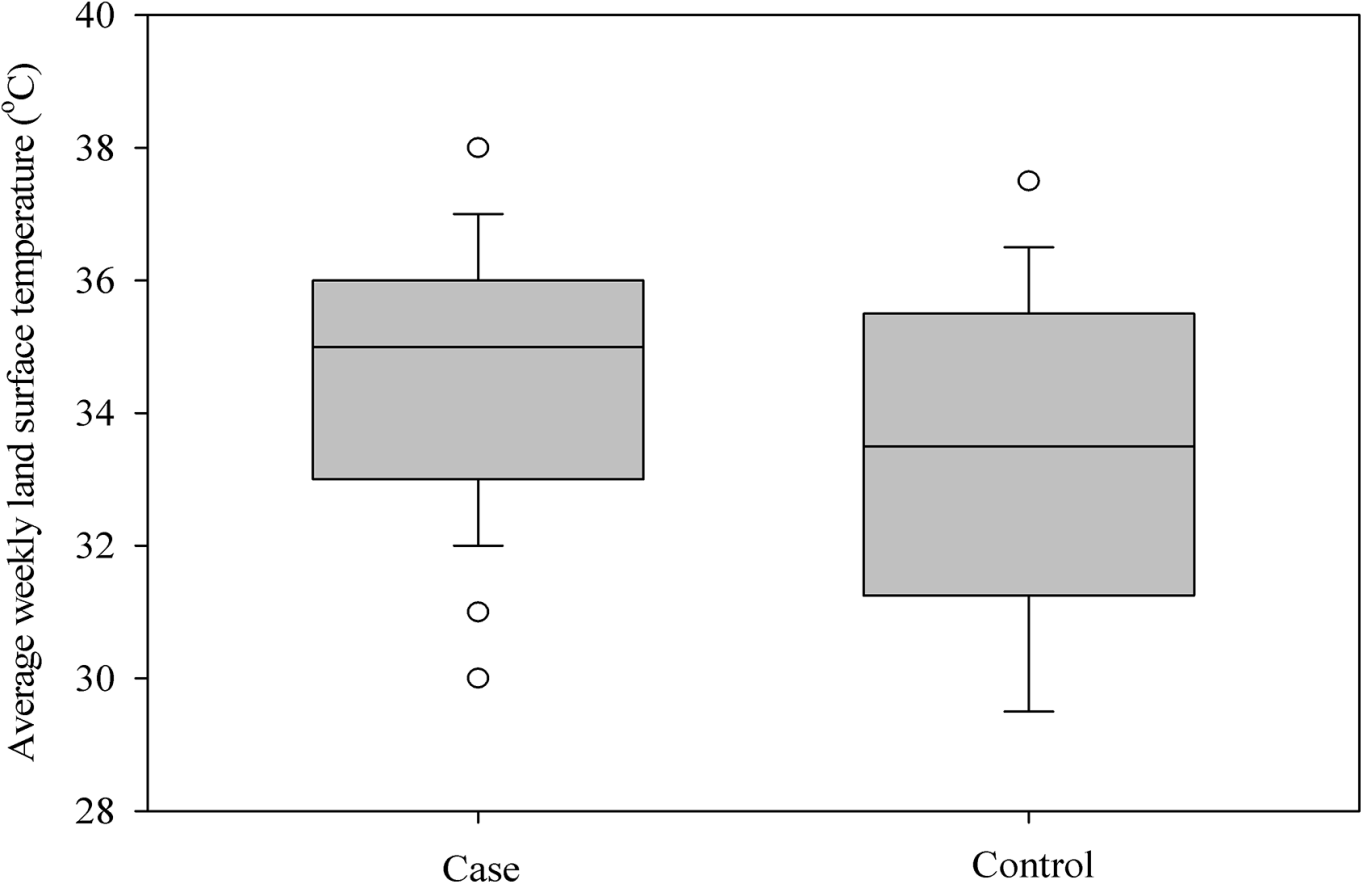
Distribution of weekly average land surface temperatures (daytime) recorded 8–12 weeks prior to diagnosis within a 2.5 km buffer area surrounding case-control locations.

The observed spatial distribution of *C*. *pseudotuberculosis* infection in Kansas based on VHC and KSVDL medical records is present in Fig 1. Most cases recorded during the study period were reported during the fall of 2012 (Sep–Nov) (Fig 2A and 2B), indicating that the infection had occurred during the preceding summer months or late spring in the region. From the univariate, deterministic, non-spatial logistic models, five variables retained their statistical significance and others were discarded. Higher soil moisture levels had a protective effect (*OR* = 1.22, 95% *CI* =1.08, 1.30) on *C*. *pseudotuberculosis* infection status in horses; and, mixed forests (*OR* = 1.18, 95% *CI* = 1.04, 1.12), grassland/herbaceous cover (*OR* = 1.22, 95% *CI* = 1.08, 1.30), total edge contrast index (*OR* = 1.33, 95% *CI* = 1.17, 2.21), and day time land surface temperature (≥35°*C*) (*OR* = 2.28 95% *CI* = 2.14, 3.31) had significantly increased the odds of infection status in horses. Of these variables, mixed forest and grassland/herbaceous cover did not retain significance in the Bayesian geostatistical model with covariates, and, individual host factor covariates (age, sex and breed) were not significantly associated with case status when they were inserted one at a time. Box plots showing differences in statistical distribution of significant univariate variables and those retained in the Bayesian geostatistical model surrounding case and control locations are present in Figs 3 and 4, respectively.

The Bayesian geostatistical model without any covariates had a DIC value of 740 and the value of the decay parameter for spatial correlation (*ϕ*) was 0.3, indicating that a high degree of spatial correlation of *C*. *pseudotuberculosis* infection prevalence was evident between locations with relatively large distances separating them. From the Bayesian geostatistical model which included all covariates, the removal of mixed forest and grassland/herbaceous cover one at a time did not significantly affect the model performance, but removing the remaining three covariates one at a time resulted in ≥10% increase to the DIC value and were therefore retained (Table 3). The final covariate model had a lower DIC value (681) relative to the model without any covariates, indicating that the presence of covariates accounted for additional spatial autocorrelation in the infection status among horses. The decay parameter for spatial correlation (*ϕ*), was still 0.3. Inclusion of host factors (age, gender and breed) did not change the posterior parameter estimates by ≥10%. The posterior distributions for all parameters followed a normal distribution pattern, and $\left(\frac{MCE}{SD}\right)$ values for individual covariates revealed no convergence issues within the burn-in period and no mixing issues were noted.

**Table 3:** Odds ratios and 95% Bayes *CrI* from Bayesian geostatistical analysis.

Model/variable	Coefficient, posterior median (95% Bayes credible interval)	Odds ratio, posterior median (95% Bayes credible interval)
a) ***Bayesian geostatistical model*, *no covariates*:**		
*α* (intercept)	1.51 (-0.42–3.16)	-
*κ* (smoothing parameter)	0.72 (0.32–1.15)	-
*ϕ* (decay of spatial correlation)	0.38 (0.11–0.82)	-
DIC	740	-
b) ***Bayesian geostatistical model with covariates*:**		
*α* (intercept)	2.01 (1.71–2.80)	-
Soil moisture		0.53 (0.25, 0.71)
*Daytime lands surface temperature*		
≥ 35°*C*		2.81 (2.21, 3.85)
28−34.9°*C*		1.15 (0.98, 2.24)
24.9−27.9°*C*		1.07 (0.59, 1.17)
≤ 25°*C*	Reference category	
Total edge contrast index		1.31 (1.27, 2.22)
*κ* (smoothing parameter)	0.81 (0.32–1.10)	-
*ϕ* (decay of spatial correlation)	0.30 (0.11–0.67)	-
DIC	681	-

The smoothing parameter, *κ* in Bayesian spatial models controls the amount of spatial continuity with distance in the *S*_*i*_ term. The decay parameter, *ϕ* refers to the decay of spatial correlation in terms of distance measured in decimal degrees (1 decimal degree at the equator is approximately 120 kilometers). A Bayesian geostatistical model with all covariates selected in the univariate procedure had a DIC value of 684, and the removal of mixed forest and grassland herbaceous cover one at a time resulted in model DIC values of 679 and 681, respectively and were discarded. The removal of Daytime LST, TECI and soil moisture one at a time in that order resulted in DIC values of 694, 691 and 703, respectively and were retained in the final model. Age and sex covariates were inserted in the Bayesian geostatistical model with covariates, one at a time, and ≥10% changes to posterior median estimates was not noted following insertions. The ‘unknown’ groups for age and sex were used as reference categories.

## Discussion

Physical environment and climatological factors influenced *C*. *pseudotuberculosis* infections among horses in the study region; and, based on the spatial correlation estimate, it appears highly likely that pigeon fever infections in the study region were influenced by common factors. As in previous studies [1], [8], seasonality in case arrivals was noted in this study, most occurring during late summer and fall months and are likely due to infections acquired in the spring and early summer. Infection with *C*. *pseudotuberculosis* has been noted to be common among young adult horses [9] but like in the present study gender and breed were not significantly associated with case status in prior studies. The lack of significant pigeon fever association with a horse’s age in this study is similar to Hall et al., [25] in their epidemiological investigation of an outbreak in 1997 in Western Colorado. It is likely that horses of all ages are equally susceptible to *C*. *pseudotuberculosis* under outbreak conditions in naïve populations.

Non-spatial models used for analyzing data with inherent spatial structure (as in this study) can lead to biased regression parameters, underestimated standard errors, narrow confidence intervals and overestimation of parameter coefficients [26], [27]. We therefore used a generalized linear geostatistical model in a Bayesian framework to account for spatial autocorrelation and to incorporate uncertainties in the input data and model parameters. This approach likely provided more accurate parameter and significance estimates and increased predictive accuracy [10], [28], [29]. Cases of pigeon fever recorded over several years (2005–2013) were modeled in this study; however, most of the cases occurred during the 2012–2013 period spread over approximately four month period. Despite this, temporal or spatio-temporal interaction effects was not noted in the disease data during the initial screening of variables. This could be because of single mode in the outbreak data and the relatively wide-spread occurrence over a four month period.

Soil is a reservoir for *C*. *pseudotuberculosis* and at least one of its biovars, *equi* has recently been shown to survive in different soil types under wide ranging environmental (humidity, ambient temperature) conditions [30]. In the present study, areas with soil that can hold higher moisture levels had a protective effect from *C*. *pseudotuberculosis* infection for horses, and conceivably this effect is similar for horses in other regions as well. Soil types that retain higher levels of moisture typically have higher clay content, and are perhaps covered by vegetation and permit less run-off [31]. Soil-borne bacterial ecology is highly regulated by soil physiochemical properties such as organic matter content, moisture and *p* ^*H*^ levels, the significance for many of which are currently not known for *C*. *pseudotuberculosis*. Future evaluations of such effects are worth considering.

Horses that had positive diagnosis for *C*. *pseudotuberculosis* infection in the present study had experienced weekly average land surface temperatures (*LST*) greater than 35°*C*(95°F) for at least a seven day period or more, eight to twelve weeks prior to diagnosis. Higher *LSTs* for prolonged periods of time (variable on different ground cover types) is an indicator used by meteorologists for determining drought conditions, which has long been suspected to be associated with *C*. *pseudotuberculosis* infection. The Great Plains region in general and Kansas in particular experienced prolonged drought conditions during the spring and summer months in 2012 [32], and recorded historically high temperatures combined with low rainfall [7], [33], [34], circumstances that may have favored an outbreak. More proximal causes for drought related disease incidence may include multiple factors. Soil is highly prone to wind erosion under drought conditions [35], consequently favoring inhalation of *C*. *pseudotuberculosis* containing airborne dust. Weakened immune system in horses exposed to persistently high temperatures or drought stress and any soil biochemical processes that boost *C*. *pseudotuberculosis* infectivity could also be proximal factors. Soil warming and drought-like conditions have resulted in altered microbial community characteristics and soil enzymatic activity under field conditions [36–38], and improving our understanding on any such effects regarding *C*. *pseudotuberculosis* may help devising preventive methods for this disease.

Total Edge Contrast Index (*TECI*), a measure of landscape fragmentation [19] was significantly associated with *C*. *pseudotuberculosis* infection status among horses in the present study. Horses that were exposed to highly fragmented landscapes may have received infection through biting insects inhabiting such landscapes, potentially also involving a transmission cycle that could include wildlife reservoirs for *C*. *pseudotuberculosis*. Landscape fragmentation leads to more and smaller landscape patches, increased patch isolation, decreased complexity of patch shape, and higher proportions of edge habitats [39], conditions that lead to loss of biodiversity and “dilution effect” [40], that subsequently favor arthropod-borne disease transmission to wildlife and humans [41], [42]. Insects have been incriminated as mechanical vectors for *C*. *pseudotuberculosis* [9], [43], and increased activity of flying-insects was observed by horse owners weeks prior to their horses received infection [25]. It is possible for insects to act as vectors for this pathogen from infected to susceptible hosts, some of which could also include other ungulates that inhabit fragmented landscapes. Kelly et al., 2012 [44] reported the isolation of *C*. *pseudotuberculosis* from abscess from captive elks, and this bacterium has been suggested to infect multiple hosts [45], including deer that may inhabit fragmented habitats. Therefore, a possibility for a transmission cycle involving insects and wild ruminant hosts seems likely but this premise needs further evaluation.

It is important to discuss a limitation of this study. Our study population originated from medical records maintained at two facilities, Kansas State University’s VHC and KSVDL, and not private practices or other sources in the region where horses may have been treated for *C*. *pseudotuberculosis* infection. Despite these two veterinary medical facilities being the largest in the state with a broad clientele in the region, there does exists a statistical possibility for some of our estimates and risk factors to change if horses from a larger population were to be included; a problem typical of many retrospective case-control studies that can only be fully mitigated by conducting carefully designed prospective sampling. Future, more robust studies are therefore necessary to better understand pigeon fever outbreaks and ecoclimatic drivers of this disease.

## Conclusions

Infection with *C*. *pseudotuberculosis* occurs in horses of all ages, gender and breed in the study region, and this disease is driven by environmental (soil moisture, landscape fragmentation) and hot and dry climatic conditions (*LST* ≥ 35°*C*). The identification of these risk factors for pigeon fever is important in the context of disease prevention. Also, it is noteworthy that soil moisture and land surface temperature are climate-change indexes that are projected to change under different IPCC (Intergovernmental Panel on Climate Change) scenarios as soon as next decade. Studies on the implication of such changes for the ecology and evolution *C*. *pseudotuberculosis* and similar soil-borne bacteria that have animal and public health significance are needed.
